# Supplementary material for: Demonstration of Three True Random Number Generator Circuits Using Memristor Created Entropy and Commercial Off-the-Shelf Components
Source: Entropy (Basel). 2021 Mar 20;23(3):371. doi: 10.3390/e23030371 (PMC8003698; doi:10.3390/e23030371)
Supplement: Supplementary file 1 [file entropy-23-00371-s001.zip › S1.docx]

Supplemental Material for “Demonstration of Three True Random Number Generator Circuits Using Memristor Created Entropy and Commercial Off-the-Shelf Components,” by Scott Stoller and Kristy A. Campbell

PERL Script for conversion of collected data to binary format.

#!/usr/bin/perl

**use** strict**;**

**use** warnings**;**

# Usage: perl csv_bin_to_datastream_von_neumann.pl inputfile1.csv inputfile2.csv output.bin

# CSV files need to be the following format example saved form Digilent AD2:

#

# #Digilent WaveForms Logic Analyzer Bus

# #Device Name: Discovery2

# #Serial Number: SN:210321A968E8

# #Date Time: 2020-11-02 00:47:01.960

# Time (s),Data

# 12496.1665,0

# 12496.1675,0

# 12496.1685,1

# 12496.1695,1

# 12496.1705,0

# 12496.1715,1

# 12496.1725,0

#

**die** "Not enough arguments. Script requires a list of input files and one output file. Input files should be .csv format and output file should be .bin." **if** @ARGV **<** 2**;**

**my** $outfile **=** $ARGV**[-**1**];**

**my** @infiles **=** @ARGV**;**

@infiles **=** **splice(** @infiles**,** 0**,** **-**1 **);**

**die** "Error: Specificed output file is not .bin file" **unless** $outfile **=~** '.bin'**;**

# fix issue where csv data file is overwritten when I forget to give a unique output file name.

**foreach** **my** $infile **(**@infiles**){**

**die** "ERROR: Infile is same as outfile.\n" **if** $infile **eq** $outfile**;**

**}**

**open(my** $FO**,** '>:raw'**,** $outfile**)** **or** **die** "Could not open outfile $outfile $!"**;**

# Variables for manipulating random sequence

**my** $num1 **=** 0**;**

**my** $count **=** 0**;**

**my** $out **=** 0x00**;**

**my** $pos **=** 0**;**

**my** $numlines **=** 0**;**

**my** $fc **=** 0**;**

# variables for deserializing stuff

**my** $b0 **=** 0**;**

**my** $b1 **=** 0**;**

**my** $c **=** 0**;**

# Iterate over all input files and convert to binary and single output file

**foreach** **my** $infile **(**@infiles**)**

**{**

**open(my** $FI**,** '<'**,** $infile**)** **or** **die** "Could not open infile $infile $!"**;**

**while(my** $line **=** <$FI>**)** **{**

**last** **if** $line **=~** ',Data'**;**

**}**

$fc**++;**

**print** **(**"Working on file $infile\n"**);**

**while(my** $line **=** <$FI>**)** **{**

**if(**$c **==** 0**)** **{**

**if(**$line **=~** ',1'**)** **{**

$b0 **=** 1**;**

**}**

**else** **{**

$b0 **=** 0**;**

**}**

$c **=** 1**;**

**}**

**else** **{**

**if(**$line **=~** ',1'**)** **{**

$b1 **=** 1**;**

**}**

**else** **{**

$b1 **=** 0**;**

**}**

$c **=** 0**;**

# only put a bit in the sequence in B0 and B1 are different

**if(**$b0 **!=** $b1**)** **{**

$out **=** $out **+** **(**$b0 **<<** $pos**);**

$pos **=** $pos **+** 1**;**

**}**

# If an entire 8-bit number has been generated print it out and count the 1's and 0's

**if(**$pos **==** 8**)** **{**

**print** $FO **pack(**'C'**,** $out**);**

# printf("0x%2x\n",$out);

$count**++;**

**if((**$out **&** 0x01**)** **==** 0x01**){**$num1**++;}**

**if((**$out **&** 0x02**)** **==** 0x02**){**$num1**++;}**

**if((**$out **&** 0x04**)** **==** 0x04**){**$num1**++;}**

**if((**$out **&** 0x08**)** **==** 0x08**){**$num1**++;}**

**if((**$out **&** 0x10**)** **==** 0x10**){**$num1**++;}**

**if((**$out **&** 0x20**)** **==** 0x20**){**$num1**++;}**

**if((**$out **&** 0x40**)** **==** 0x40**){**$num1**++;}**

**if((**$out **&** 0x80**)** **==** 0x80**){**$num1**++;}**

$pos **=** 0**;**

$out **=** 0**;**

**}**

**}**

**}**

$numlines **+=** $.**;**

**print** "$. lines Parsed!\n"**;**

**close** $FI**;**

**}**

**my** $numbits **=** $count ***** 8**;** # total number of bits found

**my** $num0 **=** $numbits **-** $num1**;** # number of zeroes

**my** $p0 **=** 100 ***** $num0 **/** $numbits**;** # percent zeroes

**my** $p1 **=** 100 ***** $num1 **/** $numbits**;** # percent ones

**my** $et **=** **time** **-** $^T**;** # elapsed time the script ran

**if(**$et **==** 0**)** **{**$et **=** 1**};** # Make sure elapsed time is not 0!

**my** $lps **=** $numlines**/**$et**;** # lines per second

**print** "\nFinished parsing all files in $et seconds!\n"**;**

**print** "Parsing a total of $numlines lines or $lps lines per second!\n"**;**

**print** "File size is $count bytes or $numbits bits\n"**;**

**print** "Found $num0 0's and $num1 1's\n"**;**

**print** "$p0 percent 0's and $p1 percent 1's\n\n"**;**

**close** $FO**;**

**exit(**0**);**
